# Comparative analysis of the complete mitogenome of *Geoffroea decorticans*: a native tree surviving in the Atacama Desert

**DOI:** 10.3389/fgene.2023.1226052

**Published:** 2023-08-10

**Authors:** Roberto Contreras-Díaz, Felipe S. Carevic, Liesbeth van den Brink

**Affiliations:** ^1^ Núcleo Milenio de Ecología Histórica Aplicada para los Bosques Áridos (AFOREST), CRIDESAT, Universidad de Atacama, Copiapó, Chile; ^2^ Laboratorio de Ecología Vegetal, Facultad de Recursos Naturales Renovables, Núcleo Milenio de Ecología Histórica Aplicada para los Bosques Áridos (AFOREST), Universidad Arturo Prat, Iquique, Chile; ^3^ Institute of Evolution and Ecology, Plant Ecology Group, Universität Tübingen, Tübingen, Germany; ^4^ Departamento de Botánica, Facultad de Ciencias Naturales y Oceanográficas, ECOBIOSIS, Universidad de Concepción, Concepción, Chile

**Keywords:** Atacama Desert, *Geoffroea decorticans*, mitochondrial genome, stress tolerance, fabaceae, extremophiles

## Abstract

Chañar (*Geoffroea decorticans* (Gill., ex Hook. & Arn.) Burkart) has been highly significant for indigenous people in the Atacama Desert for over 3,000 years. Through evolutionary processes, the *G. decorticans* mitogenome likely underwent changes facilitating its adaptation to the extreme conditions of the Atacama Desert. Here, we compare the mitochondrial genome of *G. decorticans* with those of other *Papilionoideae* family species. The complete mitogenome of *G. decorticans* was sequenced and assembled, making it the first in the genus *Geoffroea*. The mitogenome contained 383,963 base pairs, consisting of 33 protein coding genes, 21 transfer RNA genes, and 3 ribosomal RNA genes. The Chañar mitogenome is relatively compact, and has two intact genes (*sdh4* and *nad1*) which were not observed in most other species. Additionally, Chañar possessed the highest amount of mitochondrial DNA of plastid origin among angiosperm species. The phylogenetic analysis of the mitogenomes of Chañar and 12 other taxa displayed a high level of consistency in taxonomic classification, when compared to those of the plastid genome. *Atp8* was subjected to positive selection, while the *ccmFc* and *rps1* were subjected to neutral selection. This study provides valuable information regarding its ability to survive the extreme environmental conditions of the Atacama Desert.

## 1 Introduction

Chañar, *Geoffroea decorticans* (Gill., ex Hook. & Arn.) Burkart, is considered to have been one of the most important wild trees for the indigenous populations that resided in the Atacama Desert around 1000 years BP (Ugalde et al., 2021). In the present day, this species is recognized for its diverse utility as a food resource, furniture material and medicinal product (Giménez, 2004; Nuñez et al., 2009; Costamagna et al., 2013; Jiménez-Aspee et al., 2017; Cotabarren et al., 2020). Surviving and providing sustenance to local communities under such challenging conditions is a remarkable achievement for any plant. The Atacama Desert, known as the world`s oldest and driest desert, presents extreme environmental conditions including high levels of UV radiation, high temperatures, extreme aridity, and highly saline and oxidizing soils (Eshel et al., 2021; Azua-Bustos et al., 2022). *Geoffroea decorticans* also inhabits other arid and semi-arid regions in Bolivia, Peru, and Argentina (Contreras Díaz, Porcile Saavedra and Aguayo Cruces, 2018), which are facing increasing aridity due to climate change. Drought, salinity, and high temperatures are highly important environmental factors that severely restrict plant growth and development (Krasensky and Jonak, 2012). In response to these abiotic stresses, plants employ various mechanisms, such as the production of reactive oxygen species (ROS), which can cause oxidative damage to lipids, proteins, and nucleic acids, ultimately leading to programmed cell death (Van Aken et al., 2009; Tang and Zhu, 2023).

Mitochondria play a key role in plant responses to abiotic stress (Newton et al., 2004; Liberatore et al., 2016). They are involved in energy production, metabolism, regulation of PCD, and ROS production (Tang and Zhu, 2023). Compared to plastid genomes, mitochondrial genomes demonstrate substantial variability in terms of size, structure (Smith and Keeling, 2015), and gene content (Liberatore et al., 2016). Plant mitochondrial genomes (mitogenomes) exhibit distinctive characteristics, including high rates of point mutations and structural rearrangements, genome expansion and contraction, integration of foreign DNA, gene loss, and transfer to the nuclear genome (Palmer et al., 2000; Chevigny et al., 2020). It is highly likely that the mitogenome structure of *G. decorticans* has undergone changes, through evolution, enabling its adaptation to extreme conditions, and accounting for its remarkable survival capability. Studying the genetic characteristics of plants that have adapted to these harsh conditions can contribute to the preservation of this valuable genetic resource that has sustained indigenous cultures for millennia. The objective of this study is 1) to compare the structural characteristics of the mitochondrial genome of *G. decorticans* with other species of *Papilionoideae* family species, focusing on gene content, genome size, the number of protein-coding genes with RNA editing, transfer of DNA from plastid regions, and 2) to confirm its phylogeny.

## 2 Methods

Fresh leaves of Chañar were collected near Copiapó, Chile. A subsample was stored in the Index Herbariorum of Universidad de Chile, with the voucher number EIF13815, and the rest of the leaves were used for DNA isolation, using a modified cetyltrimethylammonium bromide (CTAB) protocol (Contreras et al., 2020). The concentration of the DNA was measured using a Qubit™ 3.0 fluorometer and a Qubit™ dsDNA HS Assay Kit. To verify the integrity of the DNA, an Agilent 2100 Bioanalyzer was used, prior to sequencing. The NGS library was prepared using the TruSeq DNA LT Kit and sequencing was performed on Illumina next-generation sequencing (NGS) platforms. Paired-end sequences of 150 bp were generated for both forward (R1) and reverse (R2) reads. To filter the reads we used the Trim-Galore software (Krueger, 2019), which eliminates adapter remnants and low quality sequences (phred value <25). The SPAdes 4 software, version 3.13.0 (Bankevich et al., 2012) was used to assemble the filtered reads. Additionally, we mapped the reads back to the *G. decorticans* mitogenome assembly to visualize the read coverage, using Geneious Prime v2022.0.1 (Supplementary Figure S1, http://www.geneious.com; Kearse et al. (2012)). The annotation of the mitogenome was performed using AGORA (Jung et al., 2018) and MITOFY (Alverson et al., 2010) software. The circular map of the mitochondrial genome, along with annotation information, was generated using OrganellarGenomeDRAW (OGDRAW) (Greiner et al., 2019). The final annotated mitogenome sequence of *G. decorticans* was deposited in the NCBI GenBank, with the accession number OQ707067.

The features of the *G. decorticans* mitogenome were compared with ten closely related species in the *Papilionoideae* subfamily, i.e., *Dalbergia odorifera* T.C. Chen (MW441235), *Arachis hypogaea* L. (MW448460), *Lotus japonicus* (Regel) K. Larsen (NC_016743), *Medicago sativa* L. (ON782580), *Glycine max* (L.) Merr., 1917 (NC_020455), *Phaseolus vulgaris* L. (MK176514), *Vigna angularis* (Willd.) Ohwi & H. Ohashi (NC_021092), *Pongamia pinnata* (L.) Pierre (NC_016742), *Sophora koreensis* Nakai (NC_072933) and *Castanospermum australe* A. Cunn. & C. Fraser (MK426679). Additionally, we compared the *G. decorticans* mitogenome with mitogenomes of angiosperm-species from non-polar desert habitats (Supplementary Table S1). The length of the plastid-derived region of the mitogenome was evaluated using BLASTN (Johnson et al., 2008) with default parameters, because plant mitogenomes contain sequence elements that originate in the plastid genome (plastome), known as mitochondrial DNA of plastid origin (MIPT). Therefore, each mitogenome was used as the query *versus* a database comprising the plastomes corresponding to the species: MW672397, KX257487, NC_049008, MT571487, NC_007942, NC_002694, NC_042841, JN673818, EU196765, AP012598, and MW628966.

Thirty-three protein-coding gene (PCG) sequences, i.e., *nad1, nad2, nad3, nad4, nad4L, nad5, nad6, nad7, nad9, sdh4, cob, cox1, cox2, cox3, atp1, atp4, atp6, atp8, atp9, ccmB, ccmC, ccmFc, ccmFn, rps1, rps3, rps4, rps10, rps12, rps14, rpl5, rpl16, matR, and mttB*, were used in the phylogenetic analysis of *G. decorticans* along with the previously mentioned ten *Papilionoideae* species. In addition, two *Caesalpinioideae* species, *Leucaena trichandra* (Zucc.) Urb. (NC_039738) and *Acacia ligulata* A. Cunn. ex Benth. (NC_040998), were included as outgroups. The 33 PCG sequences were aligned separately using MAFFT v7 (Katoh and Standley, 2013) and any gaps in the alignment were trimmed using trimAl v1.4 (Capella-Gutiérrez et al., 2009). Subsequently, the sequences were concatenated with Mesquite 3.81 software (Maddison and Maddison, 2023). The analyses of the mitochondrial genomes’ 33 PCG sequences were conducted using the maximum likelihood (ML) method. We did the same for the complete plastid genome sequences, using the plastome accessions listed above (including the outgroup species accessions NC026134.2 and NC028733) in order to compare the resulting phylogenetic trees. The best-fitting nucleotide substitution model of sequence evolution, model TVM + G4, was determined using the Corrected Akaike Information Criterion (AICc) through Modeltest-NG on XSEDE (Darriba et al., 2020). The ML analyses were carried out using RAxML-HPC BlackBox v.8.1.12 (Stamatakis, 2014) with 1,000 bootstrap replicates, using the CIPRES Science Gateway v3.3 (Miller et al., 2010). Non-parametric bootstrap support (BS) values were used to measure the internal nodes of the resulting trees. The ratio of non-synonymous substitution (Ka) to synonymous substitution (Ks) was calculated for 25 PCGs of *G. decorticans* and ten *Papilionoideae* species, using the KaKs_Calculator tool 3.0 (Zhang, 2022) with the MA model, where Ka/Ks values of >1 signify that the gene is subjected to positive selection, Ka/Ks values equal to 1 indicate neutral selection, and Ka/Ks values < 1 signify purification.

## 3 Results and discussion

**FIGURE 1:**
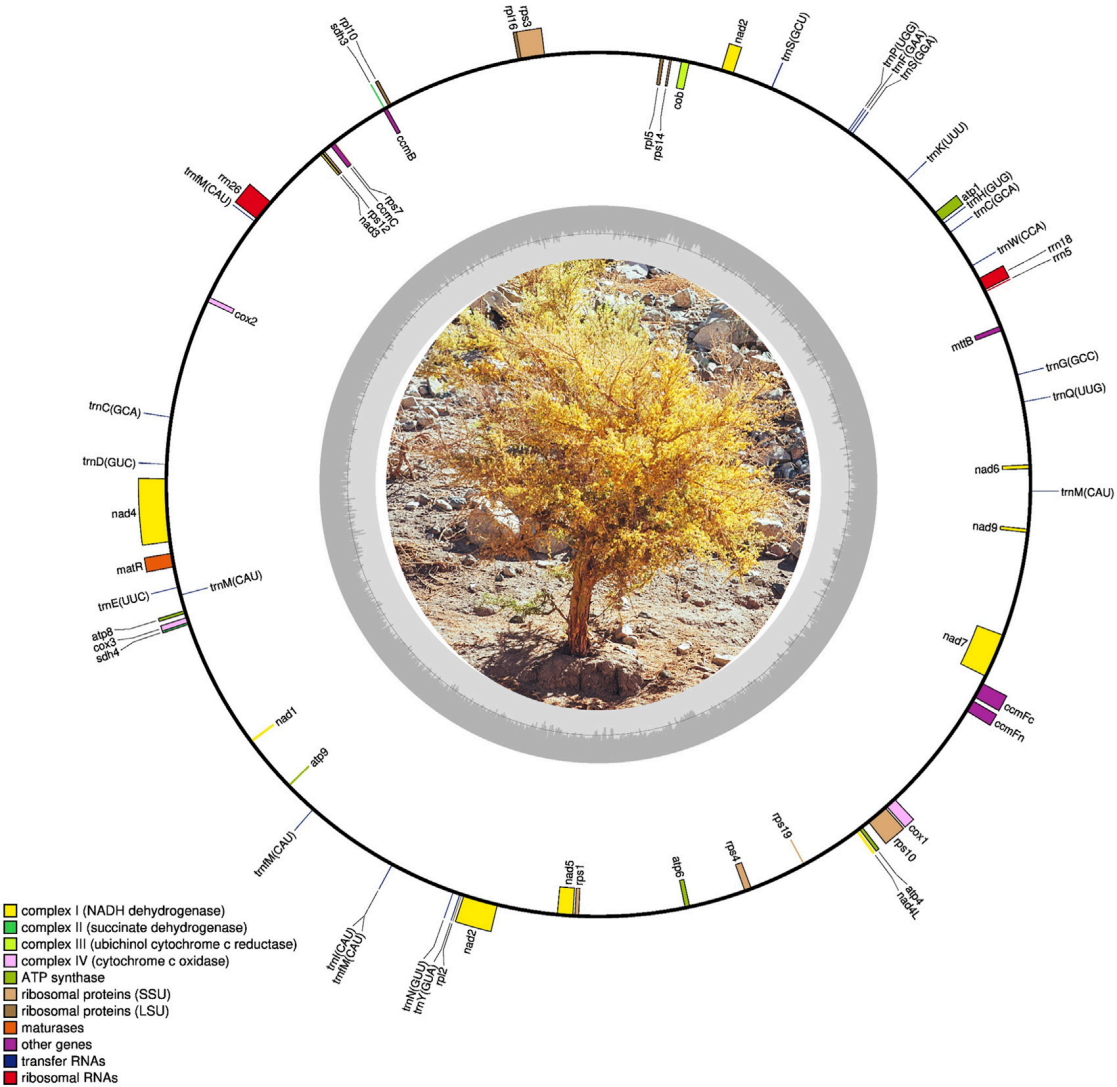
Mitochondrial maps of *Geoffroea decorticans* (size: 383,969 bp).

We successfully sequenced and assembled the complete mitogenome of *G. decorticans*, resulting in a single circular genome with a length of 383,969 bp (Figure 1; GenBank accession number OQ707067). The mitogenome sizes of *G. decorticans* and ten other *Papilionoideae* species varied from 290,285 to 592,341 bp (Table 1). Mitogenome sizes can exhibit significant variation among plant species, for example, among angiosperm the mitogenome sizes range from 66 kb in the parasitic plant, *Viscum scurruloideum* (Skippingtona et al., 2015) to 11,300 kb in *Silene conica* (Sloan et al., 2012). According to Choi et al. (2019), the median size of seed plant mitogenomes is 476 kb. However, within the *Fabaceae* family, mitogenome sizes vary considerably from 271,618 to 729,504 bp. Therefore, *G. decorticans* possesses a relatively small mitogenome size compared to other *Papilionoideae* species, but it falls within an intermediate range when compared to angiosperm mitogenomes. The variations in mitogenome size among plant species can be attributed to various factors. Mitogenomic chromosome loss, gain of exogenous DNA through intracellular gene transfer and horizontal gene transfer, and the acquisition of repetitive DNA are likely explanations for the increases and decreases observed in mitogenome sizes in angiosperms (Choi et al., 2019). Additionally, some studies suggest that changes in mitochondrial genome size can be influenced by environmental stresses (Xiong et al., 2022).

**TABLE 1:** General features of mitogenome of *Geoffroea decorticans* and other ten *Papilionoideae* species.

Species (subfamily *Papilionoideae*)	Genome length (bp)	GC (%)	Genes (*)	tRNA (**)	rRNA (**)	Protein-coding genes (PCG) (**)	Genes with mutation in first start codon (GM)	Pseudogenes	Missing genes	Exclusive gene	MIPT (bp) (***)	References
*Geoffroea decorticans*	383,969	45.3	57	21	3	30	rps4, nad4L, rps10	rpl10, sdh3, rps7, rpl2, rps19	rps2, rps11, rps13	sdh4, nad1	50,224 (13%)	This study
*Arachis hypogaea*	592,341	44.7	56	21	3	29	nad1, nad4L, rps10	rpl10, sdh4, rps19	sdh3, rps7, rpl2, rps2, rps11, rps13		33,237 (5%)	Choi et al. (2021)
*Dalbergia odorifera*	435,224	45.1	54	17	4	28	nad4L, rps10, rps14, mttB, cob	—	rpl10, sdh3, rps19, rps7, rpl2, rps2, rps11, rps13	sdh4, nad1	17,588 (4%)	Hong et al. (2021)
*Lotus japonicus*	380,861	45.4	57	20	3	31	nad1, nad4L, rps10	rpl10, sdh3, rps7, rps19, nad6, atp6, sdh4, cob	rpl2, rps2, rps11, rps13	—	12,039 (3%)	Kazakoff et al. (2012)
*Medicago sativa*	290,285	45.3	54	18	3	32	nad1	—	rpl10, sdh3, sdh4, rps19, rps7, rpl2, rps2, rps11, rps13	—	1,583 (0.5%)	—
*Glycine max*	402,558	45.0	58	19	3	30	nad1, nad4L-1, nad4L-2 rps10, ccmFc, mttB	—	rpl10, sdh3, sdh4, rps19, rps7, rpl2, rps2, rps11, rps13	—	5,507 (1,3%)	Chang et al., 2013
*Phaseolus vulgaris*	395,516	45.1	52	18	3	26	nad1, nad4L, rps10, ccmFc, mttB	—	rpl10, sdh3, sdh4, rps19, rps7, rpl2, rps2, rps11, rps13	—	3,092 (0.7%)	—
*Vigna angularis*	404,466	45.2	45	16	3	21	nad1, nad4L, mttB, ccmFc, rps10		rpl10, sdh3, sdh4, rps19, rps7, rpl2 cox2, rps2, rps11, rps13	—	4,205 (1.0%)	Naito et al. (2013)
*Pongamia pinnata*	425,718	45.0	64	24	3	32	nad1, nad4L, rps10, cox2, mttB	rps19, sdh4, rpl2, nad6, rps7	rpl10, rps2, rps11, rps13	—	7,338 (1.7%)	Kazakoff et al. (2012)
*Sophora koreensis*	519,841	44.5	60	19	3	37	nad1	sdh4	rpl10, rps19, rps2, rps11, rps13	rpl2, rps7, sdh3	53,781 (10.3%)	—
*Castanospermum australe*	542,079	45.3	58	18	3	33	nad1, rps4, rps10, mttB	rps19	rpl10, sdh3, rps7, rpl2, rps2, rps11, rps13	sdh4	2,586 (0.4%)	Zhang et al. (2019)

(*) tRNA + rRNA + PCG + GM; (**) intact genes; (***) all DNA sequences of plastid were considered.

The total GC content of *G. decorticans* was 45.3%, which was similar to the other *Papilionoideae* species, ranging from 44.5% in *S. koreensis* to 45.4% in *L. japonicus* (Table 1). In the mitogenome of *G. decorticans*, we identified a total of 57 genes, including 33 protein-coding genes (PCG), of which 30 were intact PCGs and 3 had mutations in the first start codon (Table 1). Additionally, there were 21 tRNA genes and 3 rRNA genes (Table 1). The number of genes in the mitogenomes of other *Papilionoideae* species varied from 45 genes in *V. angularis* to 64 genes in *P. pinnata* (Table 1). Moreover, *V. angularis* had the lowest number of PCGs (21) and *S. koreensis* the highest (37). The number of tRNA genes ranged from 16 in *V. angularis* to 24 in *P. pinnata*, while the number of rRNA genes was consistent across most mitogenomes (3 genes), except for *Dalbergia odorifera* which had 4 rRNA genes (Table 1). Interestingly, *G. decorticans* and *A. hypogaea* had the second-highest number of tRNA (21) genes. In angiosperms, mitochondrial tRNA genes are known to be heterogeneous, with a variable number of native tRNA genes (typically 11–13 genes) and tRNAs acquired from different sources through intracellular and horizontal transfers (Warren et al., 2021). Several studies have suggested a link between highly accelerated rates of mitochondrial sequence evolution and a reduced number of tRNA genes. For example, species like *Silene conica* and *Silene noctiflora* and *Viscum* (mistletoe) have a reduced tRNA gene content (Skippingtona et al., 2015; Warren et al., 2021). In these cases, tRNA genes are replaced by nuclear-encoded homologs, leading to a gene substitution process (Warren et al., 2021). Therefore, considering the high number of tRNA genes (21) in *G. decorticans*, it could be hypothesized that this species exhibits a reduced rate of mitochondrial sequence evolution.

The presence of genes with mutations in the first start codon was observed in the mitogenomes of the other *Papilionoideae* species, ranging from 1 gene in *Medicago sativa* and *S. koreensis* to 6 genes in *G.* max (Table 1). These mutations, known as RNA editing (C-to-U RNA editing) occur at protein genes’ first and second codon positions. The functional significance of RNA editing is not yet fully understood (Sloan et al., 2012), but it might play a role in the maintenance and function of gene and genome architecture (Linch, 2007), as well as in gene regulation, protein isoform generation and modification of active protein complexes (Lo Giudice et al., 2019). Furthermore, Murayama et al. (2012) suggested that mitochondrial function, specifically RNA editing at the *nad4* gene, interacts with and regulates the action of stress-related hormones in plants. It was found that an RNA editing site in mitochondrial *nad4* transcripts was targeted by AHG11, resulting in the production of more mRNAs for oxidative stress-responsive genes (Murayama et al., 2012). In *G. decorticans*, as well as in the other species belonging to the *Papilionoideae* family, the *nad4* gene remains intact, while in most of them the *nad4L* gene undergoes RNA editing.

Generally, vascular plants have been found to contain between 20 and 40 protein-coding genes (PCGs) in their mitogenomes (Møller et al., 2021). Mitogenomes of the species of the *Fabaceae* family have around 30 intact PCGs (Choi et al., 2019). In the case of the *G. decorticans* mitogenome, we discovered 30 intact PCGs, 3 PCGs with mutations in the first start codon, 5 pseudogenes (*rpl10, sdh3, rps7, rpl2,* and *rps19*) and 3 lost ribosomal protein genes (*rps2, rps11,* and *rps13*) (Table 1). The number of PCGs in *G. decorticans* (30) falls thus within the expected range for the *Fabaceae* species. It has been observed before that pseudogenes, truncations and deletions of the *rps7, rps11, rps13,* and *rps2* genes were prevalent in numerous *Fabaceae* species (Choi et al., 2019). This observation aligns with our findings in *G. decorticans* and the other *Papilionoideae* mitogenomes studied, except for *S. koreensis*, which retained an intact *rps7* gene. The *rps19* gene was missing in all *Papilionoideae* species used in our study (Table 1). Similarly, Wang et al. (2023) reported that most *rps* genes (*rps2*, *rps7*, *rps10*, *rps11*, and *rps19*) were absent in the mitogenome of *Photinia serratifolia*, as well as in some *Rosacea* species. The loss of ribosomal protein genes (*rps* genes) and the occurrence of putative mutations in the first start codon (RNA editing) can potentially be compensated for by nuclear genes (Newton et al., 2004). In fact, nuclear genes have the ability to influence the organization of mitochondrial genomes and regulate the expression of mitochondrial genes (Newton et al., 2004). Gene loss can occur through the transfer of a gene to the nucleus, functional substitution by a related protein, or loss of the protein and its function (Adams et al., 2002). In several *Fabaceae* species, the presence or absence of genes such as *cox2*, *rpl2*, *rpl10*, *rps1*, *sdh4,* and *sdh3* has been found to be variable (Choi et al., 2019). This variability in gene presence or absence was also observed in the eleven *Papilionoideae* species analyzed in our study (Table 1), where some species retained the genes while others exhibited pseudogenization or complete loss.

Interestingly, we discovered four *Papilionoideae* species that retained the intact *sdh* genes: *D. odorifera* (*sdh4*), *Castanospermum australe* (*sdh4*), *G. decorticans* (*sdh4*) and *S. koreensis* (*sdh3*) (Table 1). In contrast, a study by Choi et al. (2019) revealed that all *Papilionoideae* species had lost the *rpl10*, *sdh3,* and *sdh4* genes. The exclusive conservation of functional *sdh4* or *sdh3* genes, without RNA editing, such as in *G. decorticans*, may provide an important advantage for survival in the extreme conditions of the Atacama Desert. Research has demonstrated that succinate dehydrogenase (SDH) can activate the expression of stress-related genes, thereby inducing antioxidant responses and stress tolerance in plants (Jardim-Messeder et al., 2015). The authors suggested that SDH plays a crucial role in reactive oxygen species (ROS) production and in regulating both plant development and responses to stress (Jardim-Messeder et al., 2015). It is worth noting that within angiosperms, mitochondrial *rps* genes (16 genes) and *sdh* genes (*sdh3* and *sdh4*) have been lost from the mitochondrial genome multiple times throughout plant evolution (Adams et al., 2002). This further underscores the significance of intact genes in certain plant species.

On the other hand, we found two *Papilionoideae* species, *D. odorifera* and *G. decorticans*, that have the intact *nad1* gene (Table 1). Similar to what was explained earlier, this gene might play a crucial role in buffering the stress conditions experienced by *G. decorticans* on the Atacama Desert. In fact, a study by Jethva et al. (2023) investigated the function of alternative NADH dehydrogenases (*nad1*) and confirmed that this gene is essential in preventing excessive ROS formation in mitochondria during reoxygenation. The absence of *nad1* and *nad2* led to elevated ROS production, while their overexpression limited ROS levels (Jethva et al., 2023).

Plastid-to-mitochondria transfers have been suggested to have been occurring since the colonization of land by plants. Mitochondrial DNA of plastid origin (MIPT) is present in angiosperm mitogenomes in varying amounts, representing 0.1%–10.3% of the mitogenome (Sloan and Wu, 2014). In our comparative analysis, we found that the percentage coverage of MIPTs ranged from 0.4% in *C. australe* to 13% in *G. decorticans* (Table 1). It is surprising to note that *G. decorticans* exhibits higher MIPT coverage than any other angiosperm species. Initially, we had doubts regarding the accuracy of our MIPT coverage values. However, when comparing our findings, such as the 1.3% coverage in *G. max*, with the results of other studies such as Gandini and Sanchez-Puerta, (2017), we found consistency in the values. This provides confidence in the reliability of our data. In the past MIPTs were considered as “junk” sequences and were thought to have no functional contribution to the mitogenome (Wang et al., 2007). However, recent research has revealed their significance in mitochondrial function. For instance, rice MIPTs have been found to possess promoter sequences that are utilized by the mitochondrial gene *atp9* (Nakazono et al., 1996), and tRNA genes of MIPTs have also been found to contribute functionally to the mitogenome (Wang et al., 2012). The unusually high percentage of MIPTs found in *G. decorticans* may suggest a substantial acquisition of genes that could play important roles in mitogenome functioning. Investigating these genes and their potential contributes in future research would be highly valuable.

The mitogenomes from other angiosperm species that inhabit non-polar deserts (Table 2) varied between 339,352 and 758.210 bp. The mitogenomes contained 51 to 70 genes, 12 to 30 tRNA genes and 0.8%–10.3% MIPT, and were comparable to the mitogenome of the species in Table 1. Therefore, we did not find a common pattern that characterizes the mitogenomes of species that are able to inhabit deserts. Interestingly, the majority of the mitogenomes of the species from the desert contain an intact *sdh4* gene as is observed in *G. decorticans*, with the exception of *Phoenix dactylifera*, *Vigna unguiculata* and *Glycyrrhiza glabra* where the gene is lost or present as a pseudogene. As stated before, the *sdh4* gene plays an important role in the response to environmental stress. We therefore stress the importance of gaining more insight in why this gene is retained in most of the angiosperms that inhabit deserts. We observed that RNA editing had occurred in *nad1* gene of the majority of the angiosperms from deserts, however, *R. stricta* (an extremophile plant from the desert in South-West Asia) still had the intact gene, similar as *G. decorticans*. *Rhazya stricta*, as *G. decorticans*, is able to survive high temperatures and high salinity (Hajrah et al., 2017). We therefore recommend to evaluate the *nad1* gene in species along salinity gradients.

**TABLE 2:** General features of mitogenomes of angiosperm-species from non-polar desert habitats.

Species	Genome length (bp)	GC (%)	Genes (*)	tRNA (**)	rRNA (**)	Protein-coding genes (PCG) (**)	Genes with mutation in first start codon (GM)	Pseudogenes	Missing genes	Exclusive gene	MIPT (bp) (***)	References
*Rhazya stricta*	548,608	43.7	53	12	3	36	atp6, rps10	sdh3	rps2, rps11	nad1	32,810 (6%)	Park et al. (2014)
*Neltuma glandulosa*	758,210	44.8	58	19	3	34	nad1, rps10	rps7	sdh3, rps2, rps11, rps13		12,296 (1.6%)	Choi et al. (2021)
*Phoenix dactylifera*	715,001	45.1	70	30	3	35	nad1, nad4L	—	rpl10, rps10, sdh3, sdh4	rps2	73,645 (10.3%)	Fang et al. (2012)
*Tylosema esculentum*	399,572	44.7	53	15	3	31	nad1, nad4L, mttB, rps10	rps7, rps19	rpl2, rps2, rps11, rps13		20,205 (5%)	Li and Cullis (2021)
*Ceratonia siliqua*	475,642	45.3	62	21	4	34	nad1, nad4L, rps10	rps7	rps2, rps11, rps13		14,126 (2.9%)	Choi et al. (2021)
*Vigna unguiculata*	383,314	45.1	51	17	3	28	nad1, nad4L, rps10	rps19, sdh4	rpl10, cox2, rps7, rpl2, rps2, rps11, rps13, sdh3		3,122 (0.8%)	Choi et al. (2021)
*Glycyrrhiza glabra*	440,064	45.2	55	20	3	29	nad1, nad4L, rps10	rpl10, rps19, rps7, sdh4	rpl2, rps2, rps11, rps13, sdh3		5,688 (1.3%)	Choi et al. (2021)
*Haematoxylum brasiletto*	631,094	44.9	65	24	4	32	nad1, atp6, nad4L, mttB, rps10	rps7, rps13	rps2, rps11		19,949 (3.1%)	Choi et al. (2019)
*Ammopiptanthus nanus*	339,352	45.1	52	16	3	30	nad1, nad4L, rps10	rpl10, rps19, rps7	rpl2, rps2, rps11, rps13, sdh3		28,069 (8.3%)	Feng et al. (2019)
*Acacia ligulata*	698,138	45.0	59	20	3	34	nad1, rps10	—	rps2, rps7, rps11, rps13, sdh3		62,850 (9%)	Sanchez-Puerta et al. (2019)

(*) tRNA + rRNA + PCG + GM; (**) intact genes; (***) all DNA sequences of plastid were considered.

**FIGURE 2:**
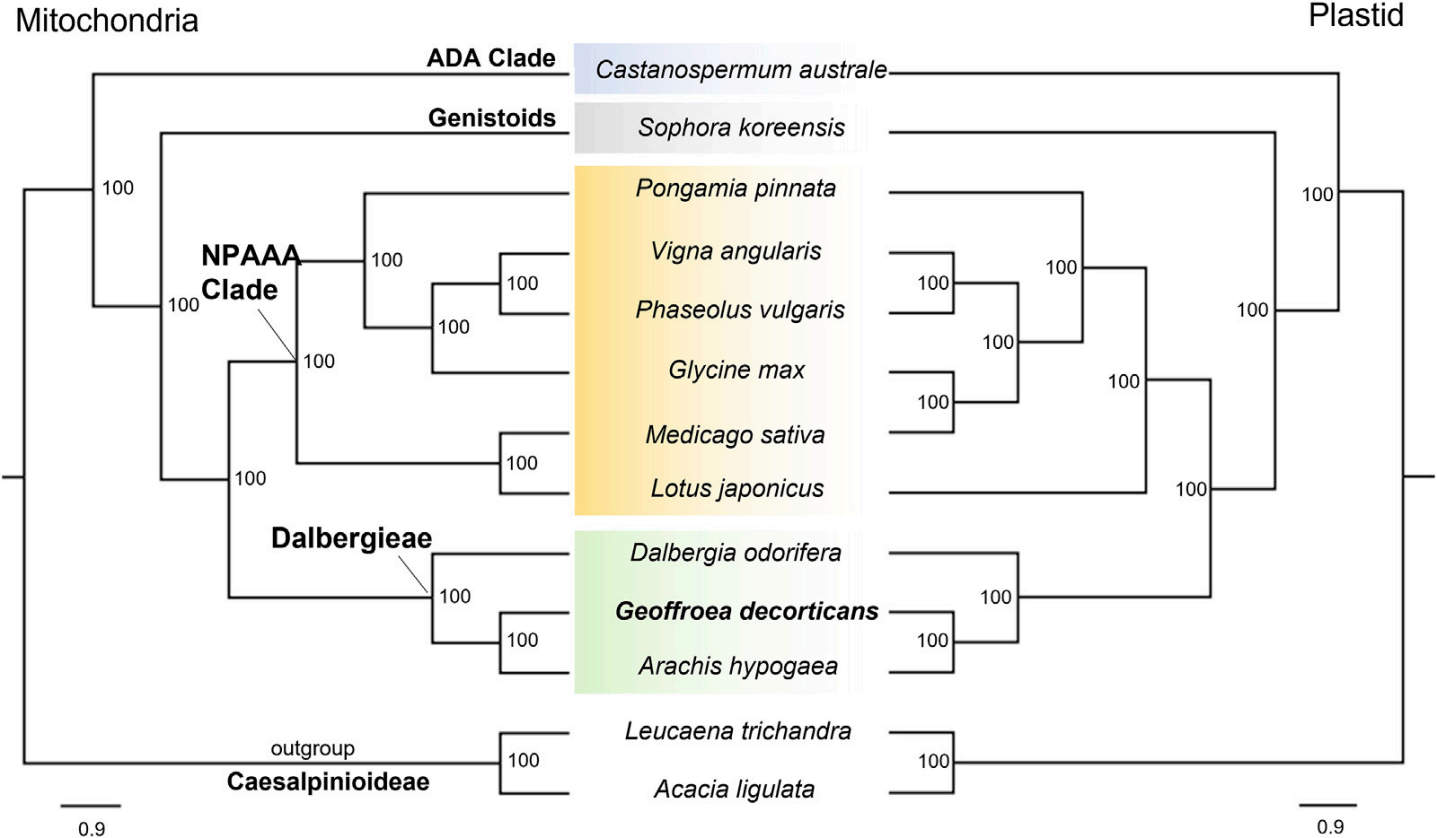
Maximum likelihood phylogeny of thirteen *Fabaceae* mitogenome based on nucleotide datasets of 33 protein-coding genes (left), and with plastid genomes (right). Bootstrap values are place on the nodes. Scale indicates number of nucleotide substitutions per site.

Previous studies have used plastid genome data to determine the molecular phylogeny and position of the genus *Geoffroea* Jack, including *Geoffroea spinosa* and *G. decorticans* (Lee et al., 2021; Contreras-Díaz et al., 2022). Additionally, researchers have developed SSR markers specific for *G. decorticans* to study the phylogeny and diversity of populations (Contreras et al., 2019; Contreras Díaz et al., 2021). However, the phylogenetic relationships of *G. decorticans* had not been assessed using mitogenome data. To address this, we analyzed concatenated sequences from 33 PCGs and complete plastid genome sequences, which were used in ML phylogenetic analysis. The resulting ML tree revealed two main clades: one containing the outgroup species *L. trichandra* and *A. ligulata* (*Caesalpinioideae*), and the other containing all 11 *Papilionoideae* species. Both clades were strongly supported with a bootstrap value of 100 (Figure 2). Within the *Papilionoideae* cluster, four subclades were identified: the *Dalbergieae* clade consisting of *A. hypogaea*, *G. decorticans* and *D. odorifera* (BP = 100); the *NPAAA* (non-protein–amino-acid-accumulating) clade including *L. japonicus*, *M. sativa*, *G. max*, *P. vulgaris*, *V. angularis* and *P. pinnata* (BP = 100); the *Genistoids* clade, represented solely by *S. koreensis* (BP = 100); and the *ADA* (*Angylocalyceae*, *Dipterygeae*, and *Amburaneae*) clade, which solely comprised *C. australe* (BP = 100) (Figure 2). These results align with previous phylogenetic studies (Cardoso et al., 2013; Choi et al., 2022). Within the *Dalbergieae* clade, two subclades were observed: one containing *D. odorifera* and the other containing *A. hypogaea* and *G. decorticans* (BP = 100) (Figure 2). This analysis strongly supported *G. decorticans* as a sister species of *A. hypogaea* (BP = 100) (Figure 2). These two species belong within the *Pterocarpus* clade, while *D. odorifera* belongs within the *Dalbergia* clade (Cardoso et al., 2013). Our phylogenetic analysis using the mitogenome database was backed up by the analysis using the plastid genome database (Figure 2), confirming the taxonomic classification of *G. decorticans*. Phylogenetic analysis of *Fabaceae* species, along with other angiosperms suggests that in certain legumes the presence of *rpl2*, *rps19*, and *sdh3* genes can be attributed to remnants of a native ancestral gene (Choi et al., 2019). In our study, we found intact *sdh4* and *nad1* genes only in *G. decorticans* and *D. odorifera* but not in *A. hypogaea*. Although these two species do not belong to the same *Pterocarpus* clade (Contreras-Díaz et al., 2022), it is possible that these intact genes have been preserved from a common native ancestor.

**FIGURE 3:**
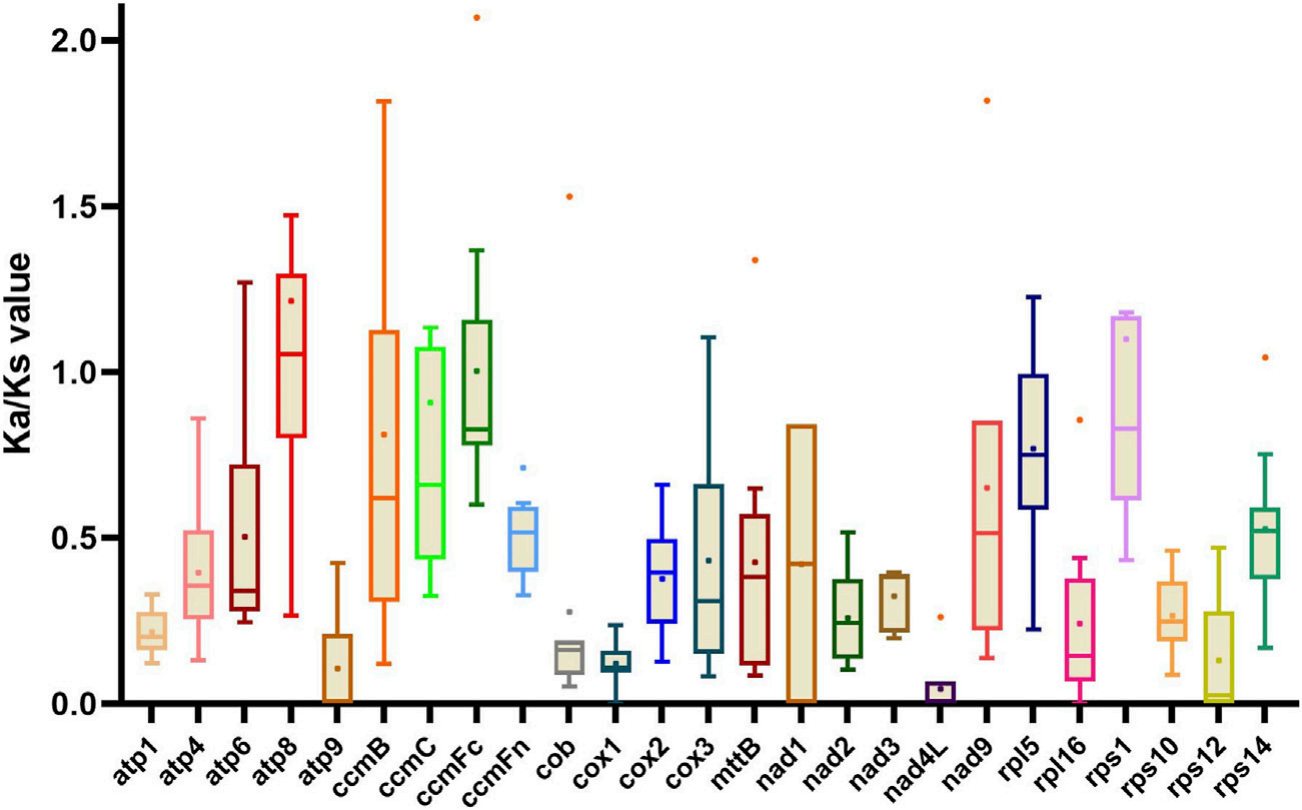
Box-and-whisker plots of Ka/Ks value of 25 protein-coding genes in *G. decorticans* and ten *Papilionoideae* species. Each box (with whiskers) shows the variation of the Ka/Ks values of a gene, among the 11 species studied using *G. decorticans* as a reference. Box plots show the median (central line), mean (dot on the box plot) and outliers.

Ka/Ks ratios can be used to reflect the natural selective pressure of protein-coding genes during evolution (Feng et al., 2019). We compared the Ka/Ks ratio for 25 protein-coding genes in the mitogenomes, comparing *G. decorticans* and the ten *Papilionoideae* species that were used in our phylogenetic analysis (Figure 3). The mean Ka/Ks value in most protein-coding genes was less than 1 (Figure 3), suggesting that these genes are purified to keep the genes functional and remove deleterious mutations. However, the mean Ka/Ks value of *atp8* (1.41) was greater than 1, (Figure 3), indicating that this gene was subjected to positive selection. Similarly, Ka/Ks values greater than 1 in the *atp8* gene were founded in the xerophytic legume species, *Ammopiptanthus mongolicus* (sister of *Ammopiptanthus nanus*) from the desert in northwest China (Feng et al., 2019); and the authors of this study have speculated that the *atp8* gene might play a role in the adaptation to dry environments. Furthermore, in the same study the evaluation of the mitogenome of *A. mongolicus* showed that the *sdh4* gene was found to be intact and unaltered (similar to *G. decorticans*), while in other legumes the gene was lost or pseudogenized (Feng et al., 2019). Further research is needed to understand why these two legume species (*G. decorticans* and *A. mongolicus*) from deserts on different continents show similar positive selection of some genes (*atp8*) and retention of other genes (such as *sdh4*).

## 4 Conclusion

Phylogenetic analysis conducted using the mitogenomes of Chañar and 12 other taxa revealed a remarkable level of consistency in taxonomic classification. When compared to other *Papilionoideae* species, the structure of the *Geoffroea decorticans* mitogenome exhibited minimal changes in terms of gene content, genome size and functional genes. However, it is important to note that the mitogenome of *G. decorticans* displayed distinct rearrangements, directionality, and organization in comparison to the other *Papilionoideae* species. One notable aspect is the conservation of native mitochondrial DNA in *G. decorticans*, as shown by positive selection for some genes, such as *atp8*, during evolution. The retention of the intact *sdh4*, *nad1* and *nad4* genes in *G. decorticans* suggests they might be important in drought tolerance mechanisms, and therefore in the species’ ability to cope with arid environments, as they have been lost in many plants that grow under more favorable conditions. Furthermore, Chañar stands out for possessing the highest amount of mitochondrial DNA of plastid origin (MIPTs) identified in any known mitogenome to date. MIPTs are involved in mitogenome functionality, and their abundance in Chañar is likely a result of the species’ evolutionary adaptation to the extreme environmental conditions of the Atacama Desert. The acquisition of additional DNA from other organelles, such as plastids, through horizontal gene transfer, provides Chañar with unique genetic material that potentially contributes to its survival strategies. The combination of conserved genes that facilitate drought stress responses and the acquisition of plastid material has likely contributed to the exceptional characteristics of *G. decorticans*. This species not only survives, but also provides sustenance to the inhabitants of the driest desert on Earth, making it an example of adaptation in challenging environments.

## Data Availability

The datasets presented in this study can be found in online repositories. The names of the repository/repositories and accession number(s) can be found below: https://www.ncbi.nlm.nih.gov/nuccore/OQ707067.1/. The raw reads have been deposited in NCBI SRA with the number “PRJNA719569”.
