# Supplementary material for: Comparative analysis of the complete mitogenome of Geoffroea decorticans: a native tree surviving in the Atacama Desert
Source: Front Genet. 2023 Aug 10;14:1226052. doi: 10.3389/fgene.2023.1226052 (PMC10448962; doi:10.3389/fgene.2023.1226052)
Supplement: Supplementary file 1 [file DataSheet1.docx]

**Supplementary Materials**


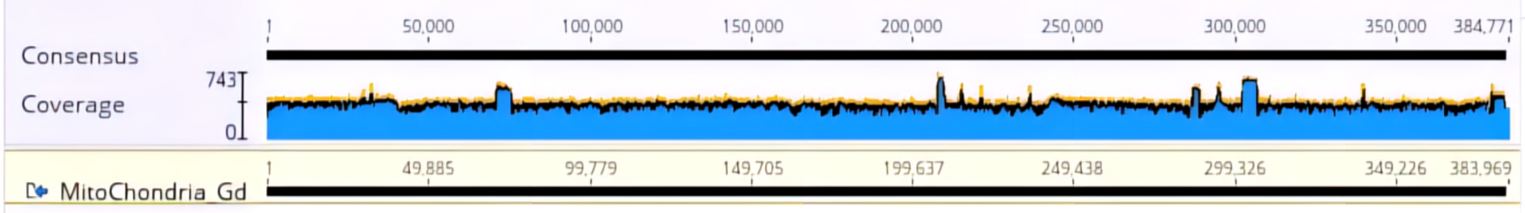


**Figure S1**. Mitochondrial DNA sequencing coverage. The number of reads (depth of coverage) for each nucleotide position are showed. All peaks correspond to mitochondrial DNA of plastid origin MIPT, although some MIPT had lower coverage.

**Table S1**. Mitogenomes of extremophile plant species currently available in GenBank NCBI

| **Species** | **Classification** | **Locality, environmental or stress context** | **Mitogenome available (accessions)** | **Plastid genome accessions used to calculate MIPT** | **Reference** |
| --- | --- | --- | --- | --- | --- |
| *Anastatica hierochuntica* | Angiosperm | Negev Desert | No |  | Bechtold et al 2018(*) |
| *Eutrema salsugineum* | Angiosperm | Yukon territory, Canada | No |  | Bechtold et al 2018 |
| *Rhazya stricta* | Angiosperm | Bahrah (Saudi Arabia) | NC_024293 | NC_024292 | Bechtold et al 2018 |
| *Schrenkiella parvulum* | Angiosperm | Salt flats in Tuz (Turkey) | No |  | Bechtold et al 2018 |
| *Craterostigma plantagineum* | Angiosperm | South Africa | No |  | Bechtold et al 2018 |
| *Sporobolus stapfianus* | Angiosperm | South Africa | No |  | Bechtold et al 2018 |
| *Sporobolus pyramidalis* | Angiosperm | Yemen Africa | No |  | Bechtold et al 2018 |
| *Vincetoxicum mongolicum* | Angiosperm | Ningxia, China | No |  | Bechtold et al 2018 |
| *Haberlea rhodopensis* | Angiosperm | Balkan mountains, Bulgaria | MH757117 |  | Bechtold et al 2018 |
| *Zygophyllum xanthoxylum* | Angiosperm | Desert areas in China and  Mongolia | No |  | Bechtold et al 2018 |
| *Populus euphratica* | Angiosperm | Shapotou Desert Experiment  and Research, Ningxia, China | No |  | Bechtold et al 2018 |
| *Calotropis procera* | Angiosperm | Saudi Arabia | No |  | Bechtold et al 2018 |
| *Pohlia nutans* | Mosses | Antarctica | NC_046778 |  | Boulc’h et al 2020(**) |
| *Colobanthus quitensis* | Angiosperm | Antarctica | No |  | Boulc’h et al 2020 |
| *Deschampsia antarctica* | Angiosperm | Antarctica | No |  | Boulc’h et al 2020 |
| *Populus euphratica* | Angiosperm | Desert | No |  | Boulc’h et al 2020 |
| *Physcomitrium patens* | Mosses | Desiccation | NC_007945 |  | Boulc’h et al 2020 |
| *Craterostigma plantagineum* | Angiosperm | Desiccation | No |  | Boulc’h et al 2020 |
| *Arabidopsis lyrata* | Angiosperm | Drought | No |  | Boulc’h et al 2020 |
| *Solanum commersonii* | Angiosperm | Freezing | No |  | Boulc’h et al 2020 |
| *Chorispora bungeana* | Angiosperm | High altitude | No |  | Boulc’h et al 2020 |
| *Crucihimalaya himalaica* | Angiosperm | High altitude | No |  | Boulc’h et al 2020 |
| *Hippophae rhamnoides* | Angiosperm | High altitude | No |  | Boulc’h et al 2020 |
| *Arabidopsis halleri* | Angiosperm | High metal | No |  | Boulc’h et al 2020 |
| *Mesembryanthemum crystallinum* | Angiosperm | High salt | No |  | Boulc’h et al 2020 |
| *Oryza coarctata* | Angiosperm | High salt | No |  | Boulc’h et al 2020 |
| *Salicornia europaea* | Angiosperm | High salt | No |  | Boulc’h et al 2020 |
| *Sporobolus virginicus* | Angiosperm | High salt | No |  | Boulc’h et al 2020 |
| *Suaeda maritima* | Angiosperm | High salt | No |  | Boulc’h et al 2020 |
| *Heritiera littoralis* | Angiosperm | Mangroves | No |  | Boulc’h et al 2020 |
| *Saxifraga longifolia* | Angiosperm | Mountains | No |  | Boulc’h et al 2020 |
| *Oropetium thomaeum* | Angiosperm | Desert | No |  | Boulc’h et al 2020 |
| *Phoenix dactylifera* | Angiosperm | Desert | NC_016740 | NC_013991.2 | Boulc’h et al 2020 |
| *Rhizophora mangle* | Angiosperm | Mangroves | No |  | Boulc’h et al 2020 |
| *Lobularia maritima* | Angiosperm | Salt stress | No |  | Oh et al 2012(***) |
| *Amaranthus hypochondriacus* | Angiosperm | Drought | No |  | Oh et al 2012 |
| *Limonium sinense* | Angiosperm | Salt stress | No |  | Oh et al 2012 |
| *Halosarcia indica* | Angiosperm | Salt stress | No |  | Oh et al 2012 |
| *Avicennia marina* | Angiosperm | Salt stress | No |  | Oh et al 2012 |
| *Festuca rubra* | Angiosperm | Salt stress | No |  | Oh et al 2012 |
| *Spartina alterniflora* | Angiosperm | Salt stress | No |  | Oh et al 2012 |
| *Neltuma glandulosa* | Angiosperms | Desert of southern Texas; Chihuahuan desert, Mexico | MW448450 -  MW448455 | KJ468101 | Choi et al 2021 |
| *Tylosema esculentum* | Angiosperms | Kalahari Desert | OK638188,OK638189 | NC_067756 | Li and Cullis 2021 |
| *Ceratonia siliqua* | Angiosperms | Desert, Israel, Siria | MW448447 -  MW448449 | NC_047061 | Choi et al 2021 |
| *Vigna unguiculata* | Angiosperms | Desert, Mexico | MW448464 | NC_018051 | Choi et al 2021 |
| *Glycyrrhiza glabra* | Angiosperms | North Africa; drought stress | MW448466 | NC_024038 | Choi et al 2021 |
| *Haematoxylum brasiletto* | Angiosperms | Sonora Desert, Mexico | NC_045040 | NC_047060 | Choi et al 2019 |
| *Ammopiptanthus nanus* | Angiosperms | Desert and arid regions of Central Asia | NC_046466 | NC_034743 | Feng et al 2019 |
| *Acacia ligulata* | Angiosperms | Simpson Desert, Australia | NC_040998 | NC_026134.2 | Sanchez-Puerta et al 2019 |

(*) Bechtold U, et al. (2018) Plant Life in Extreme Environments: How Do You Improve Drought Tolerance? Front. Plant Sci. 9:543. doi: 10.3389/fpls.2018.00543

(**)Boulc’h P-N, et al. (2020) Abiotic stress signalling in extremophile land plants, Journal of Experimental Botany 71(19): 5771–5785, doi: 10.1093/jxb/eraa336

(***) Oh, DH, et al. (2013) Life at the extreme: lessons from the genome. Genome Biol 13:241, doi: 10.1186/gb-2012-13-3-241
